# Higher PEEP in intubated COVID-19-associated ARDS patients? We are not sure

**DOI:** 10.1186/s13054-022-04207-6

**Published:** 2022-10-25

**Authors:** Andrey I. Yaroshetskiy, Sergey N. Avdeev, Anna P. Krasnoshchekova, Galia S. Nuralieva

**Affiliations:** 1 Sechenov First Moscow State Medical University (Sechenov University), 8 bld 2, Trubetskaya Str, Moscow, Russia 119991; 2 Research Institution for Clinical Surgery Division, Anesthesiology and Critical Care Department, Pirogov Russian National Research Medical University, 1, Ostrovitianova str, Moscow, Russia 117997

## To the editor

We have read with great interest the article published in Critical Care by Somhorst et al. entitled «PEEP‑FiO_2_ table versus EIT to titrate PEEP in mechanically ventilated patients with COVID‑19‑related ARDS» [1]. This retrospective observational study aimed to select the «optimum» positive end-expiratory pressure (PEEP) in mechanically ventilated patients with COVID-19-related acute respiratory distress syndrome (ARDS) by electrical impedance tomography (EIT), based on the balance between alveolar collapse and overdistension. The authors got interesting results, showing that non-obese patients with COVID-19-related ARDS had low lung recruitability with «optimum» PEEP close to 10 cmH_2_O, and PEEP levels above the appropriate value in the high PEEP/inspiratory fraction of oxygen (FiO_2_) table in obese patients led to «significant alveolar recruitment and less alveolar overdistension». The authors found «a significant positive correlation between set PEEP and body mass index» that could be the result of PEEP selection according to high chest wall elastance (based on transpulmonary pressure measurement) [2]. The next valuable point mentioned by the authors is that the longer the time course of the disease, the lower would be the lung recruitability which apparently reflected the progression of lung injury in COVID-19 which is characterized by relatively low recruitability [3–5]. Nevertheless, we need to discuss some points concerning the study.

First of all, patients were recruited in the study during the first wave of the pandemic which was characterized by excessive use of invasive mechanical ventilation [6, 7]. The authors did not present intubation criteria and detailed description of patients’ status, such as the prevalence of non-respiratory organ dysfunction, frailty, sepsis, the duration of noninvasive ventilation (NIV) before enrollment, duration of low PaO_2_/FiO_2_ and tachypnoea (respiratory distress), and other factors that could lead to early or delayed intubation. Therefore, it is unclear to which subgroup of the population with COVID-19-ARDS the results of the study could be extrapolated.

Second, let’s focus on the oxygenation status as a stratification tool for the selection of the appropriate respiratory support method in ARDS [8]. So, according to Table 1, PaO_2_/FiO_2_ in the study by Somhorst et al. was 162 [110–201] mmHg, and the FiO_2_ level was around 50% [1]. We can speculate that at least a significant part of these patients could be oxygenated noninvasively—by NIV or high-flow oxygen therapy (HFOT). We have enough data by September 2022, based on RCTs [9, 10], observational studies outside ICU, and a meta-analysis of those studies [11] showing very high efficacy of noninvasive respiratory support in mild, moderate, and even moderate-to-severe hypoxemia in COVID-19-related acute respiratory failure (ARF) reaching 70% overall and more. These studies used moderate PEEP levels (if used at all), without intubation, deep sedation, and neuromuscular blockade and came up with the same outcomes as in the present study by Somhorst et al. So, the study did not answer the question of what PEEP value is appropriate for the cohort of invasively ventilated COVID-19-ARDS patients, in whom NIV/HFOT failed.

Third, all patients had slightly decreased compliance and low driving pressure (around 10 cmH_2_O) that did not change after the PEEP increase, probably, because of low lung recruitability and non-uniform distribution of the lung injury and atelectasis—(multi)local but not diffuse lung injury [12]. To prove this statement, one might compare EIT data and driving pressure changes during the PEEP trial in obese patients. Re-aeration of the dorsal lung units by higher PEEP levels seen on EIT images without decrease in driving pressure in obese patients apparently confirms non-homogeneous lung collapse predominantly in dependent zones. Unfortunately, lung CT scans that would shed light on the pattern of lung injury and percentage of lung involvement are lacking. Moreover, if lung recruitment was clinically significant after the PEEP increase, we might expect to see an increase in PaO_2_/FiO_2_ which was not observed.

Fourth, the authors assumed that the EIT method precisely reflects alveolar collapse and overdistension. Costa et al. described the original method of calculating overdistension, postulating that «local tidal volume can be estimated by EIT on a pixel by pixel basis, considering that it correlates very well with local impedance variations», thus local lung compliance could be calculated by dividing the local impedance change to the local driving pressure [13]. This assumption has two potential sources of misinterpretation: First, we cannot measure local driving pressure; second, in hyperinflation, the strain can increase without volume change, *i.e.*, without increase in lung impedance. Accordingly, incorrect interpretation of the EIT data can lead to lung overdistension due to inappropriately high PEEP.

Lastly, the authors followed the concept of the «optimal» individual PEEP as the combination of minimum collapse and minimum overdistension. This hypothesis never found its confirmation. Moreover, it seems that overdistension could be worse than alveolar collapse [14, 15].

We must say that the results of the study should be interpreted with caution. We can hypothesize that these results can draw the following conclusions: 1) a PEEP trial guided by EIT resulted in moderate PEEP levels in non-obese patients with COVID-19-ARF without regard to the PEEP/FiO_2_ table, and PEEP according to BMI in obese patients, which can be higher than «high» PEEP/FiO_2_ table levels; 2) these data need confirmation in a large scaled observational studies and RCTs, including NIV. Studies on advanced physiological monitoring (transpulmonary pressure, lung volumes, etc.) in COVID-19-associated ARF during invasive and noninvasive ventilation are urgently needed to confirm or reject this hypothesis.

## Data Availability

Not applicable.

## Abbreviations

ARDS: Acute respiratory distress syndrome
ARF: Acute respiratory failure
BMI: Body mass index
EIT: Electrical impedance tomography
FiO_2_: Inspiratory fraction of oxygen
HFOT: High-flow oxygen therapy
ICU: Intensive care unit
NIV: Noninvasive ventilation
PaO_2_: Partial pressure of oxygen in arterial blood
PEEP: Positive end-expiratory pressure
RCT: Randomized controlled trial
